# Aerobic Training in Patients with Congenital Myopathy

**DOI:** 10.1371/journal.pone.0146036

**Published:** 2016-01-11

**Authors:** Gitte Hedermann, Christoffer Rasmus Vissing, Karen Heje, Nicolai Preisler, Nanna Witting, John Vissing

**Affiliations:** Copenhagen Neuromuscular Center, Department of Neurology, Rigshospitalet, University of Copenhagen, Copenhagen, Denmark; University of Granada, SPAIN

## Abstract

**Introduction:**

Congenital myopathies (CM) often affect contractile proteins of the sarcomere, which could render patients susceptible to exercise-induced muscle damage. We investigated if exercise is safe and beneficial in patients with CM.

**Methods:**

Patients exercised on a stationary bike for 30 minutes, three times weekly, for 10 weeks at 70% of their maximal oxygen uptake (VO_2max_). Creatine kinase (CK) was monitored as a marker of muscle damage. VO_2max_, functional tests, and questionnaires evaluated efficacy.

**Results:**

Sixteen patients with CM were included in a controlled study. VO_2max_ increased by 14% (range, 6–25%; 95% CI 7–20; p < 0.001) in the seven patients who completed training, and tended to decrease in a non-intervention group (n = 7; change -3.5%; range, -11–3%, p = 0.083). CK levels were normal and remained stable during training. Baseline Fatigue Severity Scale scores were high, 4.9 (SE 1.9), and tended to decrease (to 4.4 (SE 1.7); p = 0.08) with training. Nine patients dropped out of the training program. Fatigue was the major single reason.

**Conclusions:**

Ten weeks of endurance training is safe and improves fitness in patients with congenital myopathies. The training did not cause sarcomeric injury, even though sarcomeric function is affected by the genetic abnormalities in most patients with CM. Severe fatigue, which characterizes patients with CM, is a limiting factor for initiating training in CM, but tends to improve in those who train.

**Trial Registration:**

The Regional Committee on Health Research Ethics of the Capital Region of Denmark H-2-2013-066 and ClinicalTrials.gov H2-2013-066

## Introduction

Congenital myopathies (CM) are genetically and histologically heterogeneous disorders caused by mutations in genes that often encode sarcomeric proteins. Traditionally, CM is divided into three subtypes defined by histopathological findings on muscle biopsy: nemaline, central core and centronuclear myopathy. Genetic studies have shown that this division cannot stand alone. Thus, each subtype, defined by histological findings, can be caused by mutations in multiple genes, and mutation in a single gene can result in multiple phenotypic and histological presentations. Despite the variations, these patients typically present with a non-progressive early-onset muscle weakness. Some patients have involvement of extraocular and/or facial musculature. Creatine kinase (CK) levels are in the normal range or slightly elevated [1–3]. Fatigue is a prominent symptom in patients with CM [4].

The majority of patients with neuromuscular disorders live a sedentary life [5]. It has become evident from several studies, conducted in the last decade, that aerobic training is beneficial for several muscle diseases [6–10]. The effect of training has never been investigated in patients with CM, and due to the involvement of contractile proteins of the sarcomere, it could be hypothesized that training is deleterious in these conditions. The aim for this study was to investigate the effect on maximal oxygen uptake (VO_2max_) of 10 weeks of aerobic training in a cohort of patients with CM.

## Materials and Methods

### Subjects

To be eligible for inclusion in the study, patients had to be over the age of 18 and had to have confirmed pathogenic mutation(s) in genes, which are known to cause CM (see Table 1).

**Table 1:** Characteristics of the sixteen patients included in the study. Patients 1–7 completed the training program. Patients 8–16 dropped out of the training program. Patients 5–11 participated in the non-intervention group. Patients 2 and 11 had bilateral foot drop requiring braces. Patient 3 took Alendronat 70 mg weekly to prevent osteoporosis, and Patient 7 used BiPAP at night. Patient 8 took Budesonid inhalations for asthma.

Patient no./sex	Affected gene	Completed training		Dropped out of training program		Non-intervention	
		Age	BMI	Age	BMI	Age	BMI
1/M	ACTA1	27	13				
2/F	NEB	40	30				
3/F	NEB	33	31				
4/F	DNM2	24	17				
5/F	DNM2	56	21			56	21
6/F	RYR1	40	19			40	19
7/M	TPM3	23	22			23	22
8/M	NEB			30	27	30	27
9/M	TPM3			31	13	31	13
10/M	DNM2			29	28	29	28
11/M	NEB			54	23	54	23
12/F	TPM2			43	18		
13/F	RYR1			42	30		
14/F	RYR1			32	18		
15/M	DNM2			28	18		
16/M	RYR1			36	21		
Mean ± SD		35 ± 12	22 ± 7	36 ± 9	22 ± 6	38 ± 13	22 ± 5

Additionally, patients had to be able to cycle for 30 minutes to be included in the study. Patients with competing medical conditions such as contractures, arthritis, heart- and lung diseases that could interfere with interpretation of exercise testing in the eyes of the investigator, and patients that performed more than one hour of aerobic training weekly were excluded.

**Fig 1:**
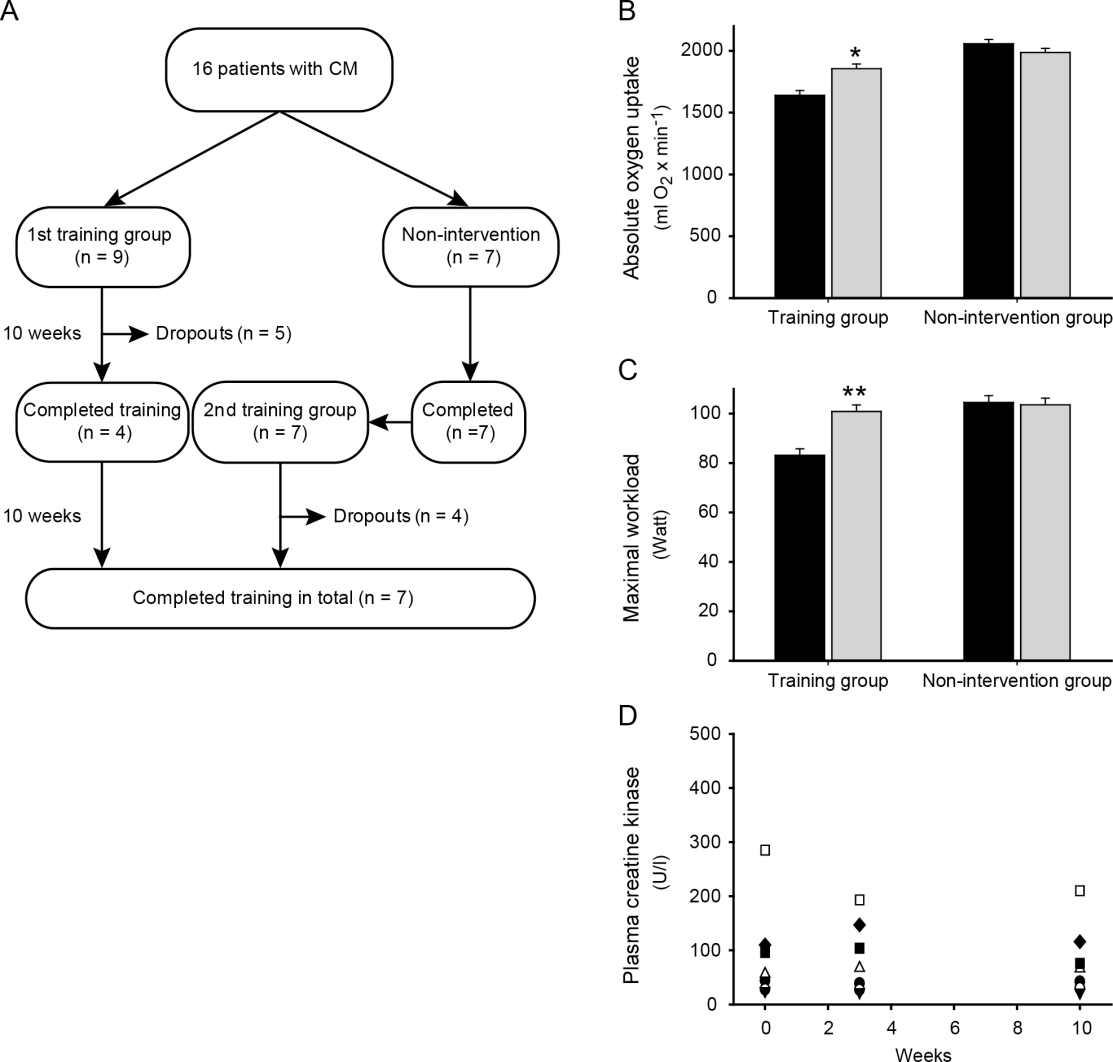
Flowchart, maximal oxygen uptake, workload and plasma creatine kinase levels. (A) 16 patients with CM were included in the study. Four patients from the 1^st^ training group completed the training program. A non-intervention group of seven CM patients were tested twice, 10 weeks apart, before they participated in the training program (2^nd^ training group). Only 3 patients from the non-intervention group completed the subsequent training program. In total, nine patients dropped out of the training program. (B) VO_2max_ before and after 10 weeks of aerobic training in seven CM patients with an improvement corresponding to 215 ml O_2_ · min^-1^ (CI 121–308 ml O_2_ · min^-1^, * p = 0.001) (left bars). VO_2max_ before and after 10 weeks of normal daily living in seven patients with CM (right bars). Black bars represent values before, and gray bars represent values after. The change seen in the intervention group was significant compared to the change in the non-intervention group (mixed Anova, p < 0.001). (C) Maximal workload before and after 10 weeks of aerobic training in seven CM patients who improved W_max_ by 18 W (CI 11–24 W, ** p = < 0.001) (left bars). W_max_ before and after 10 weeks of normal daily living in seven CM patients (right bars). Black bars represent values before, and gray bars represent values after. The change seen in the intervention group was significant compared to the change in the non-intervention group (mixed Anova, p < 0.001). (D) Dots represent plasma CK levels at week 0, 3 and 10 from the seven CM patients who finished the training program. All values are within the normal range or slightly elevated when corrected for age and gender. VO_2max_: maximal oxygen uptake; CM: congenital myopathy; W_max_: maximal workload; CK: creatine kinase.

Patients were recruited from a cohort of 33 genetically confirmed cases of CM older than 18 years. Eleven of the patients did not fulfil inclusion criteria, and 6 patients were not interested in participating. The remaining 16 patients were included in the study (Fig 1A and Table 1)

### Study design

The study setup dictated a non-blinded design, but was controlled by a parallel non-intervention group that served as a control for the trained group (see Fig 1A, flowchart). On inclusion, all patients accepted to conduct a 10-week training program. Patients were divided in two groups; one started the training program and the other started a period of unchanged daily living (non-intervention group). After 10 weeks of unchanged daily living, the patients in the non-intervention group started their training program. The allocation to training first or later was not randomized, because of time constraints for a number of participants. All patients had to complete a test day before and after the 10-week intervention or non-intervention periods. Based on effect (a change of 18.6% or more) and variation (SD = 0.15) in VO_2max_ responses to aerobic training for 10–12 weeks in other muscle diseases [6–9], and assuming a chance of type 1 error of 0.05 and a type 2 error of 0.19, the needed number in the training group to complete training is 7 persons.

### Test days

Patients performed a peak exercise test on a cycle ergometer and three functional tests; a 6-minute walk test (6MWT), a five repetition sit-to-stand test (FRSTST), and a timed 14-step-stair-test (T14SST) (tests described in Table 2 and [10]). At inclusion, muscle strength was evaluated by a modified 10-step Medical Research Council (MRC) scale. Muscle strength has been measured by handheld dynamometry in previous studies on 10–12 weeks of cycle training in other muscle diseases [11–12]. No significant outcome was seen with this type of exercise, so muscle strength was not evaluated with a dynamometer in this study. Forced vital capacity (FVC) was measured by spirometry in all patients.

**Table 2:** Results of functional tests and questionnaires from the intervention and non-intervention groups. 6MWT: 6-minute walk test (m: distance in meters). FRSTST: five times repetitive sit-to-stand test (s: seconds), the patient was asked to rise and sit from a chair five times as fast as possible. T14SSTn: timed 14-step-stair-test (normal speed), the patient had to climb and decline 14 steps at their normal speed. T14SSTq: timed 14-step-stair-test (quick), the patient had to climb and decline 14 steps as fast as possible. FSS: fatigue severity scale. ND: not determined. Values are mean ± standard deviation. P-values in the right column are calculated by a mixed Anova test, no significant difference was found between the improvements seen in the intervention group compared with the improvements in the non-intervention group.

	Intervention group (n = 7)				Non-intervention group (n = 7)				Mixed ANOVA test
	Before	After	% improvement	p-value	Before	After	% improvement	p-value	p-value
6MWT (m)	460 ± 97	462 ± 83	0.9	0.827	491 ± 66	479 ± 70	-2.7	0.061	0.118
FRSTST (s)	18.3 ± 7.9	17.0 ± 10.2	10.6	0.311	14.6 ± 4.1	14.8 ± 4.2	-1.4	0.866	0.326
T14SST_n_ (s)	26.6 ± 12.1	29.4 ± 19.3	-4.9	0.361	20.3 ± 4.3	21.3 ± 3.8	-6.4	0.339	0.553
T14SST_q_ (s)	23.3 ± 13.3	27.1 ± 21.6	-9.4	0.290	16.5 ± 5.6	16.6 ± 5.1	-2.2	0.776	0.290
SF36 Fatigue	51 ± 19	54 ± 23	7.0	0.570	58 ± 19	59 ± 16	2.5	0.846	0.820
FSS score	4.9 ± 1.9	4.4 ± 1.7	10.4	0.083	4.5 ± 1.5	ND			

Patients completed four questions from the SF-36 questionnaire concerning fatigue on all test days.

Furthermore, they completed the Fatigue Severity Scale (FSS) at baseline and after the training. FSS rates fatigue perceived in the preceding week. FSS score ≥ 4 indicates abnormal fatigue, and a score ≥ 5 indicates severe fatigue [4].

Plasma creatine kinase (CK) levels were measured on test days and after three weeks of training as a marker of exercise-induced muscle damage.

### Peak exercise testing

Testing was performed on an electronically braked Lode Excalibur Sport cycle ergometer. The person who measured VO_2max_ was not blinded, but the tests were carried out systematically after standard guidelines in the laboratory. Prior to testing, patients were fitted with a heart rate monitor, 3-lead ECG electrodes and a facial mask. Pulmonary gas exchange was measured with breath-by-breath indirect calorimetry (Quark CPET, Cosmed Srl, Milan, Italy). Workload was increased incrementally. The patients were verbally encouraged to keep exercising for as long as possible. The test was terminated when the patients were unable to maintain their pedaling cadence (10% drop in rotation per minute (RPM) in 20 s). The VO_2max_ was a mean of the period of 20 seconds where they performed the highest VO_2max_.

### Home-based, pulse-monitored training

The patients trained at home on a stationary bike three times weekly for ten weeks. The patients were provided with stationary bikes and instructed in the set-up of the bike. It was not important that they trained on a specific type of stationary bike. They were instructed to have a small flexion of 5–15 degrees in their knee when they sat on the bike with the leg stretched. They were advised to cycle with the same cadence as used at the peak exercise test, between 60–80 RPM. The intensity was controlled by a pulse interval corresponding to 70% of their VO_2max_ as determined from the peak exercise test. The duration of the training sessions increased from 20 minutes during the first two weeks to 30 minutes the last eight weeks. The training was always initiated by a 5-10-minute warm-up to allow the patients to reach their pulse interval gradually before starting the training session.

Compliance was monitored by downloading data from pulse watches. The patients also kept a diary of their training sessions and recorded adverse effects. Patients were phoned weekly to facilitate compliance.

### Primary outcome measures

Primary efficacy was change in VO_2max_ and fatigue (FSS questionnaire). VO_2max_ is an indicator of cardiovascular fitness and aerobic endurance, and a change reflects the physiological response to training. Patients were not told prior to testing that training might improve their fatigue.

### Secondary outcome measures

Secondary efficacy was assessed by changes in 6MWT, FRSTST, and T14SST.

### Ethics and statistics

The Regional Committee on Health Research Ethics of the Capital Region of Denmark approved the study (H-2-2013-066), and written informed consent was obtained from all patients.

Values are mean ± standard error, unless otherwise stated. Changes were assessed by a paired Student t-test, and a p-value < 0.05 (two tailed testing) was considered significant. Some results, when indicated, were assessed by a mixed Anova test.

## Results

### Demographic data of the patients

The included patients represent patients with CM, who have mutations in a broad range of genes (Table 1). The mean BMI for the patients was normal (Table 1). Two patients had a BMI of 13, which is not an uncommon finding in patients with CM [2].

### Compliance and dropouts

Due to a high dropout rate, only seven of sixteen patients finished the training program (Fig 1A). Compliance was high in those who finished the training program. They completed 26–32 training sessions of 30 target sessions during the 10 weeks.

Nine patients dropped out of the training program, and fatigue was a major reason for dropping out for six of the patients. One patient dropped out due to personal matters, and two patients were lost to follow-up. None of the dropouts exercised for longer than half of the training period before exclusion and were therefore not evaluated for efficacy according to the protocol.

### Peak oxidative capacity, workload and plasma CK levels

Training caused a significant increase in VO_2max_ from 28 ± 3 to 31 ± 4 ml O_2_ · kg^-1^ · min^-1^ (range of increase, 1.9–7.1 ml O_2_ · kg^-1^ · min^-1^) (Fig 1B). Parallel changes were seen in peak workload (Fig 1C). Mean peak heart rate was 184 ± 4 before and 183 ± 2 after 10 weeks of training in the intervention group. No change was seen in the non-intervention group with an average VO_2max_ of 31 ± 3 at baseline vs. 30 ± 3 ml O_2_ · kg^-1^ · min^-1^ at 10 weeks (Fig 1B).

All patients had stable CK levels during training (before, 92 ± 34; after, 80 ± 25 U/l; p = 0.334) (Fig 1D).

### Functional tests, muscle strength, FVC and questionnaires

**Fig 2:**
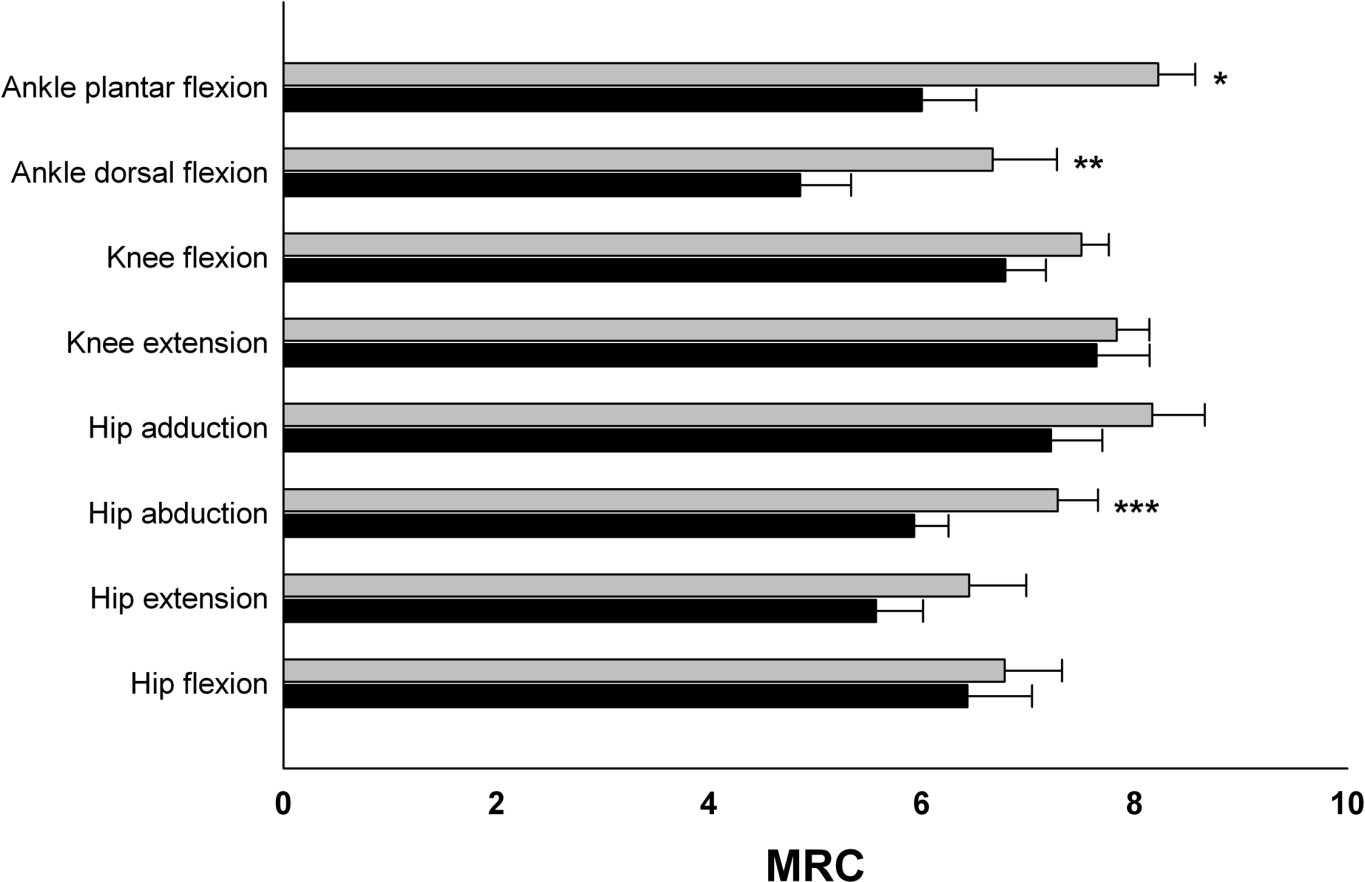
Baseline muscle strength evaluated by MRC score in the seven patients who finished the training program (black bars) and in the nine patients who dropped out of the training program (gray bars). A significant difference was found between the two groups in three muscle groups; ankle plantar flexion, * p < 0.001; ankle dorsal flexion, ** p = 0.032; hip abduction, *** p = 0.014. Error bars indicate standard error of the mean.

No changes were seen in any of the functional tests or SF36 questionnaire in either group (see Table 2). Baseline MRC scores are shown in Fig 2. FVC (n = 15) as percentage of predicted values were lower than seen in age-, sex- and height-matched healthy subjects (69 ± 5% of normal). Baseline FSS scores (n = 14) showed severe fatigue (5.1 ± 0.4), which is concordant to observations found in other CM patients [4]. FSS scores from the intervention group tended to decrease following training from 4.9 ± 1.9 to 4.4 ± 1.7 (p = 0.08).

## Discussion

This study indicates that 10 weeks of moderate-intensity, endurance training on a cycle ergometer is safe and an efficient way to improve fitness. But it is difficult to apply this therapy to all patients with CM due to fatigue. If trained, fatigue can potentially be reduced in patients with CM. Training improved VO_2max_ significantly by 14% in the seven patients who completed the training program. No change was seen in mean peak heart rate indicating that the change was independent of will of the patient trying to push harder or encouragements by the un-blinded investigator. Improving fitness in patients with CM probably has other beneficial effects, as it is known that higher levels of physical fitness delay all-cause mortality, including lower rates of cardiovascular disease and cancer [13]. This is important, since patients with neuromuscular diseases are at higher risk of developing cardiovascular risk factors as a part of metabolic syndrome [5].

Although most patients carry mutations in genes encoding sarcomeric proteins or other contraction-excitation coupling related mechanisms, exercise does not appear to cause sarcomeric injury, since CK values were stable during the intervention and patients reported no myalgia. The improvement corresponds to improvements seen in similar training programs for patients with different types of muscular dystrophies [6–10].

Training of patients with other muscle diseases has shown unchanged or reduced fatigue following training. In our study, fatigue tended to decrease with training, which is of particular interest in this group, as fatigue is a disabling symptom in the every day life of patients with CM [4]. Fatigue was also the main single cause for dropping out. Nine patients dropped out of the training program, six of them due to fatigue. Fatigue therefore poses a limitation for initiating training in patients with CM, which is unlike the experience in other muscle diseases. However, fatigue is a major contributing factor for the lack of training effects in patients with motor neuron diseases [12, 14]. Unfortunately, fatigue was not assessed over time in the non-intervention group for comparison. However, fatigue is not likely to change in such a short period of time without interventions, as CMs are largely non-progressive. Pulmonary function tests showed a rather mild impairment, and only one patient used nocturnal respiratory assistance (Table 1). No correlation was seen between the FVC and FSS scores. It is therefore unlikely that increased fatigue in our patients with CM was caused by respiratory failure. Despite weekly phone contact between the investigator and the patient, patients were not compliant.

Since CM subtypes are very rare, it was necessary to include patients with different genetic backgrounds. Training responses could differ among CM genotypes and gender. However, training had the same consistent positive effect across all patients studied, which indicates that training is beneficial and safe in most types of CM.

The three functional tests were included, which improved with a similar training program in patients with LGMD2L (anoctamin 5 deficiency) [10]. However, no significant improvements were seen in patients with CM (see Table 2). This could relate to the short duration of the trial. Likely the improvements in endurance could translate into functional improvements if the training period had been prolonged, but future studies must investigate this.

Overall, the present study shows that for those CM patients who were able to complete the training, there was a significant improvement in VO_2max_, but it also illustrates how difficult it is to maintain CM patients on a training schedule, possibly due the high basal level of fatigue in this patient group. Alternative methods of training such as strength training or shorter training sessions at higher work intensities should be explored in CM.

## Supporting Information

S1 Text Consort checklist. (DOC)

S2 Text Trial study protocol in Danish. (DOCX)

S3 Text Trial study protocol in English. (DOCX)

## Abbreviations

CM: congenital myopathy
CK: creatine kinase
FSS: Fatigue Severity Scale
FRSTST: five repetition sit-to-stand test
MRC: Medical Research Council
T14SST: timed 14-step-stair-test
VO_2max_: maximal oxygen uptake
6MWT: 6-minute walk test
