# Supplementary material for: Aerobic Training in Patients with Congenital Myopathy
Source: PLoS One. 2016 Jan 11;11(1):e0146036. doi: 10.1371/journal.pone.0146036 (PMC4709049; doi:10.1371/journal.pone.0146036)
Supplement: S2 Text — (DOCX) [file pone.0146036.s002.docx]

Forsøgsprotokol

Effekten af et cykel-træningsprogram hos patienter med muskelsvind

1. Formål og baggrund

Baggrund

Muskelsvind omfatter en lang række arvelige sygdomme, der er kendetegnet ved at patienter har et progressivt tab af muskelmasse og styrke, grundet et øget henfald af muskelceller. Det øgede tab af muskelceller, bunder i defekter i en bred vifte af proteiner med funktioner i muskelcellen.

Muskelsvindssygdomme er medfødte sygdomme, der fænotypisk ligner hinanden, idet de alle involverer skeletmuskulaturen og i større eller mindre grad fører til et fremadskridende tab af muskelstyrken. Symptomdebut og hastigheden af muskelsvindet hos patienterne kan variere fra sygdom til sygdom og indenfor den enkelte undertype af muskelsvind.

Paraklinisk set har muskelsvindspatienter forhøjede plasma CK-værdi, som tegn på fortløbende skade på muskelceller og billediagnostisk kan der ses fedtinfiltration i muskulaturen.

**Træning**

Virkningen af fysisk aktivitet hos raske såvel som kronisk syge personer, har gennem de seneste år vakt stor interesse i forskningsverden, hvor man både nationalt og internationalt har studeret i betydningen af fysisk aktivitet for syge og raske.

Træning af muskelsvindspatienter har indtil for få år siden ikke været anbefalet, da man mente det bidrog til en accelereret nedbrydning af patienternes muskler. En lang række klinisk forsøg har dog indenfor de senere år påvist at fysisk træning, og her særligt aerob konditionstræning både er en effektiv og sikker behandling af en række muskelsygdomme. Man har indtil videre ikke undersøgt effekten af konditionstræning i alle muskelsvindssygdomme, og der findes kun få studier, der har overvåget effekten af træningsprogrammer, der strækker sig over længere tid end et par måneder.

Formål

En persons maksimale iltoptagelse (VO_2max_) er et klinisk standardiseret mål for en persons kondition, defineret ved personens evne til at optage, cirkulere og forbruge ilt, og dermed evnen til at udføre et fysisk arbejde.

Vi ønsker at undersøge om et 10 ugers cykeltræningsprogram kan øge konditionsniveauet hos muskelsvindspatienter og bedre deres funktionsevne i hverdagen, og om en eventuel gavnlig effekt af træning, efterfølgende kan holdes ved lige ved fortsat træning over et år. Som noget nyt ønsker vi i dette projekt også at inkludere muskelsvindspatienter, der er kørestolsbrugere og træne dem under særlige vilkår.

Effekten af aerob træning er af særlig interesse iblandt muskelsvindspatienter, da mutationer i musklernes støtte- eller funktionelle proteiner potentielt kan føre til en øget følsomhed overfor mekanisk belastning af musklerne.

Effekten af træningsprogrammet vurderes ved de standardiserede kliniske parametre: maksimal iltoptagelse (VO_2max_), hjertefrekvens (HR_max_) og arbejdsbyrde (W_max_). Desuden måles muskelenzymet Creatin Kinase (CK) i plasma. Patienterne gennemgår før og efter træningsperioden også en muskelstyrketest, et spørgeskema omhandlende selvvurderet muskelfunktion og livskvalitet, samt en række funktionstests, omfattende 6-minutters gangtest, stol-stand-test og en trappebestigningstest. Hos gruppen af muskelsvindspatienter, der er kørestolsbrugere vil de parametre vi måler på være en smule anderledes. Effekten af træningsprogrammet vil hos dem blive målt ved forbedring i funktion af mavetarmsystemet, tendensen til siddesår og -smerter, deres aktivitetsniveau, og hvilken kraft de kan træde med ved cykling på en speciel cykel (et konfigureret W_max_ mål).

Det gælder for langt de fleste nedarvede muskelsygdomme, at ingen kurative behandlinger findes. Det vil derfor være af stor betydning, at få belyst om fysisk træning har nogen klinisk anvendelse, som behandlingsredskab hos denne gruppe af patienter.

**Primære effektmål**

- Konditionstest med bestemmelse af VO_2max_ og/eller W_max_ på en kondicykel.

**Sekundære effektmål**

- De tre funktionstests, der viser forsøgsdeltagernes funktionalitet før og efter træningsperioden.
- Muskelstyrke bestemt før og efter træningsperioden.
- Forsøgsdeltagernes CK-værdier, der fortæller noget om muskelskade før, under og efter forsøget.
- Selvvurderet forbedring i muskelfunktion
- Fald i forekomst af siddesår, balde- og rygsmerter, bedre funktion af mavetarmsystem (spørgeskema til kørestolsbrugere)

1. Metode

Forsøgsdeltagere

Forsøget udføres, som et 10-ugers langt cykel-træningsprogram i eget hjem, hvor patienter med genetisk verificeret muskelsvindssygdomme træner 30 minutter ad gangen hver anden dag mindst 3 gange om ugen.

Hvis patienterne viser sig at have en gavnlig effekt af den aerobe træning, vil de blive tilbudt, at fortsætte i et næsten identisk monitoreret træningsprogram, der løber over et år.

Forsøgsdeltagerne bliver inviteret til at deltage i forsøget pr. brev. Det forventes at forsøget vil inkludere 7-9 forsøgsdeltagere for hver underdiagnose i alderen 18-75 år. Vi planlægger at undersøge 4-6 muskelsvindssygdomme, så det vil sige 30-50 forsøgspersoner i alt. Projektet søger at inkludere personer med sygdommene Bethlem myopati, Ullrichs disease, undertyper af Limb-girdle muskeldystrofi type 2, mitokondriebetinget muskeldystrofi, samt Duchenne og Becker muskeldystrofi.

Forsøget inkluderer desuden tre-fem forsøgsdage på Neuromuskulær forskningsenheds afsnit 3342, Rigshospitalet. Der vil være to forsøgsdage før det 10 uger lange cykeltræningsprogram og en efter træningsprogrammet. Hvis den enkelte forsøgsdeltager ønsker at deltage i forsøget, der løber over et år vil det inkludere en yderligere forsøgsdag efter 6 måneder og en afsluttende forsøgsdag efter et år.

Træningsbeskrivelse for ambulante forsøgsdeltagere

**Konditionstest og cykeltræningsprogram**

Inden træningsperiodens start bestemmes forsøgsdeltagernes VO_2 max_, HR_max_ og W_max_ ved en max-test på en ergometercykel, hvor arbejdsbelastningen gradvis øges til udmattelse. Der sigtes imod at testen varer ca. 12-15 min.

Efterfølgende gennemgår forsøgsdeltagerne et 10 ugers cykeltræningsprogram i eget hjem på en udleveret ergometercykel. Forsøgspersonerne skal under forsøget cykle hver anden dag, mindst tre gange om ugen, og træningen skal foregå i et bestemt pulsinterval, der svarer til at de arbejder ved 70 % af deres maksimale iltoptagelse. Pulsintervallet monitoreres med et udleveret pulsur. Træningen optrappes i forsøget, sådan at forsøgspersonerne i første uge kører 10 minutter i det fastsatte pulsinterval, i anden uge kører i 20 minutter i pulsintervallet, og fra tredje uge og frem kører i 30 minutter i pulsintervallet. Forsøgspersonerne skal altid varme op i 5 minutter ved lav belastning før de kører i pulsintervallet.

Efter 10 ugers træning får forsøgsdeltagerne atter målt VO_2 max_, HR_max_ og W_max_ på en ergometercykel. Resultaterne fra denne test sammenlignes med resultaterne opnået i testen umiddelbart før træningsprogrammet (anden test) for eventuel forbedring/forværring. Hvis den enkelte forsøgsdeltager er interesseret i at fortsætte med at træne efter de 10 uger er overstået, kan de fortsætte med træningsprogrammet i et år, til vurdering af den langvarige effekt af aerob konditionstræning hos muskelsvindspatienter.

Forsøgsdeltagerne skal deltage i to testdage før cykeltræningsprogrammet påbegyndes for at sikre at de resultater, der opnås i forsøget ikke bunder i en læringseffekt fra test til test.

**Muskelstyrkevurdering**

Forsøgsdeltagernes muskelstyrke vurderes før og efter træningsprogrammet. Målingen foretages med et håndholdt dynamometer. Muskelstyrkemålingerne fra patienternes m. biceps brachii, m. quadriceps femoris og m. gastrocnemius sammenlignes efter forsøgets afslutning.

**6 minutters gangtest**

Patienten skal på 6 minutter gå så langt som muligt på en 30 meter lang bane, der er afmærket af to kegler. Resultat før og efter cykeltræningsprogrammet registreres.

**Timed up and down stair test**

Patienten starter 30 cm fra en trappe med 14 trin, der hver måler 19,5 cm i højden. Herfra skal de instrueres i at de hurtigt, men sikkert, skal bestige trappen, vende på toppen og returnere til bunden på tid. Patienterne kan selv vælge hvordan de bestiger trappen. Testen foretages før og efter træningsprogrammet med registrering af eventuel effekt, og er et udtryk for deres funktionelle mobilitet.

**Chair stand test**

Forsøgspersonen skal sidde på en stol uden armlæn med krydsede arme og fødderne i gulvet. Under instruktion skal patienten rejse sig fra stolen uden brug af arme/hænder. Hvis dette lykkedes skal patienten rejse sig 5 gange fra stolen på tid. Tiden registreres og tiden før cykeltræningsprogrammet sammenlignes med tiden efter. Hvis patienten ikke kan rejse sig fem gange noteres antal gange det lykkedes patienten at rejse sig.

**Spørgeskema omhandlende selvvurderede muskelfunktion**

Forsøgsdeltagere skal udfylde et spørgeskema, omhandlende deres selvvurderede muskelfunktion før og efter forsøget.

**CK-måling**

CK er en markør for graden af muskelskade hos patienterne. CK bestemmes ved blodprøvetagning på de to forsøgsdage (uge 0 og 10) på Neuromuskulær forskningsenhed og under cykeltræningsprogrammet i uge 2, 4 og 7, på Neuromuskulær forskningsenhed eller hos egen læge efter aftale om dette. Hvis forsøgsdeltagerne efterfølgende inkluderes i den del af forsøget, der strækker sig over et år, får de efterfølgende målt CK-værdier hver tredje måned.

Træningsbeskrivelse for kørestolsbundne forsøgsdeltagere

Da de muskelsvindspatienter, der er kørestolsbundne er for svage til at cykle på en konventionel ergometercykel, skal deres træning foregå på en motoriseret trædecykel (som fx Motomed Viva 2). Den motoriserede trædecykel er opbygget således at forsøgsdeltagerne træner siddende i deres egen stol eller kørestol. Cyklens pedaler kører automatisk rundt uden hjælp fra forsøgsdeltageren, men er i stand til at måle hvor stor en kraft brugeren bidrager med.

Som de andre forsøgsdeltagere skal brugerne af denne cykel deltage i tre testdage, træne i 10 uger (også pulsmonitoreret hver anden dag og 35 minutter af gangen) og have foretaget tre blodprøver efter henholdsvis 2, 4 og 7 uger. Kørestolsbundne forsøgsdeltagere kan af praktiske grunde ikke udføre de tre funktionstests eller få målt deres VO_2max_, hvorfor det endelige effektmål på træningseffekten er ændringen i hvor stor en kraft de kan træde med (W_max_).

1. Oprettelse af forskningsbiobank

Der udtages under forsøget blodprøver til bestemmelse af muskelenzymet CK. Med blodprøverne sikres det, at der ikke sker nogen muskelskade under træningsforløbet. Der udtages under hele forsøgets forløb fem blodprøver. Ved hver blodprøve udtages 5 ml blod, hvilket svarer til 1 % af hvad man afgiver ved en normal tapning, som bloddonor. Der er altid en minimal risiko for infektion ved blodprøvetagning. Dette vil blive forebygget ved afspritning af indstiksstedet. Blodet vil efter det er udtaget straks blive analyseret og en eventuel rest vil straks blive destrueret. Der oprettes derfor ingen biobank.

1. Statistiske overvejelser og styrkeberegning

De statistiske beregninger bygger på tidligere studier der har vist effekt af konditionstræning hos patienter med muskelsvind. Den følgende styrkeberegning er baseret på, at man skal kunne påvise en effekt iblandt hver enkelt muskelsvinds-undertype separat. Tallene i styrkeberegningen er baseret på de resultater vi har opnået i en række lignende træningsforsøg med muskelsvindspatienter, som vi tidligere har foretaget.

Styrkeberegning:

(z_1-α/2_ + Z_1-β_ )^2^ * SD^2^

n ≥ _________________________

d^2^

Z_1-α/2_ = 1.96; Risiko for type 1 fejl: (α=0.05)

Z_1-β_ = 1.28; Risiko for type 2 fejl: (1-β) = 90%

SD = 0,152;

d = 0,186; (MIREDIF) Forbedring i VO_2max_ fra før til efter træningsperioden.

Dvs.

(1.96 + 1.28 )^2^ * (0,152)^2^

n ≥ _________________________ = 7

(0,186)^2^

Passende deskriptiv statistik (means, SD, median og range) vil blive anvendt.

Forskelle mellem før og efter træningen vil blive testet med en parret t-test.

Yderligere relevant statistik vil anvendes efter behov.

Den del af studiet, der beskæftiger sig med de kørestolsbundne muskelsvindspatienter kan betragtes, som værende af en mere eksplorativ natur.

Ud fra styrkeberegningen søger vi at inkludere 7-9 muskelsvindspatienter, fra hver underdiagnose med et samlet antal forsøgspersoner på 50.

1. Inklusions og eksklusionskriterier

Inklusionskriterier

Danske patienter diagnosticeret med muskelsvindssygdom.

Eksklusionskriterier

Patienter, der skønnes for fysisk svage/svækkede til at kunne gennemføre 10 ugers træningsprogram.

Patienter med konkurrerende lidelser som konfunderer fortolkningen af behandlingseffekten ved fysisk træning. Heriblandt også forsøgspersoner der er gravide eller lider af hjerte-karsygdomme. Alle patienter, der ønsker at medvirke i forsøget vil før forsøgsstart være vurderet, for eventuel kardiomyopati/hjertesvigt.

Patienter som ikke mentalt kan kooperere.

Forsøgspersoner

Projektet vil inkludere forsøgspersoner i alderen 18-75 år og både kvinder og mænd kan inkluderes. Det regnes med at 7-9 forsøgspersoner bliver inkluderet per muskelsvinds-underdiagnose. I alt ca. 50 patienter.

1. Bivirkninger, risici og ulemper

I forsøget indgår en invasiv procedure i form af blodprøvetagning. Der er altid en minimal risiko for infektion, der forebygges ved afspritning af indstiksstedet. Nogle patienter bliver nervøse og ængstelige før blodprøvetagning. Dette forebygges ved grundig information samt ved at behandle patienten i afslappet evt. liggende stilling. Selve cykelforsøget indebærer risiko for udvikling af hjerteproblemer, samt evt. ubehag i forbindelse med den hårde fysiske anstrengelse for forsøgspersonerne. Derudover er der risiko for at patienterne får muskelsmerter og evt. muskelskade. Alt dette vil dog blive forsøgt forebygget ved at monitorere puls og visuel smerte/udtrættelsesskala (Borg-skala) under testen.

Der er også en ukendt risiko for muskelskade forbundet ved at træne personer med stærkt eleveret CK-værdi. Dette søges forebygget ved at informere patienterne om faretegn, som primært består af muskelsmerter efter belastning, ved at monitorere CK-niveauet under forsøget og ved at den projektansvarlige har løbende kontakt til forsøgsdeltagerne forsøget igennem. Patienterne får ligeledes udleveret en træningsvejledning, der i detaljer beskriver normale og unormale bivirkninger ved fysisk træning, samt en liste over faretegn, de skal være opmærksomme på, under og efter træning. Hvis patienterne i forsøgets løb får uacceptable bivirkninger, som følge af konditionstræningen, trækkes de omgående ud af forsøget. Patienterne kan hele forsøget igennem ringe til projektgruppen, der ved klager om ildebefindende vil tage en faglig vurdering af den enkelte patients videre forløb.

1. Respekten for forsøgspersonernes fysiske og mentale integritet samt privatlivets fred.

Oplysningerne vedrørende den enkelte forsøgsperson beskyttes efter lov om behandling af personoplysninger og sundhedsloven. Projektet anmeldes til Datatilsynet.

1. Økonomiske forhold
2. Projektet er blevet iværksat af professor John Vissing i samarbejde med stud.med. Christoffer Rasmus Vissing.
3. Projektet støttes af Rigshospitalet (daglig drift) og der søges derudover om fondsstøtte til udgifter forbundet med projektet. Støtten vil blive udbetalt til Rigshospitalet til dækning af driftsudgifter, cykler, pulsure og lignende. Videnskabsetisk komite vil blive orienteret om hvilken fondsstøtte, der opnås
4. Der foreligger ingen økonomisk interessekonflikt, og forsøgsansvarlige har ingen økonomisk eller anden tilknytning til private virksomheder, som skulle have interesser i det beskrevne projekt.
5. Relevante klausuler i kontrakt mellem sponsor og forsøgsstedet

Professor John Vissing er forsøgsansvarlig. Der er ingen relevante klausuler

1. Vederlag eller andre ydelser

Der tilbydes frokost til alle forsøgspersoner efter afslutning af testdagene og forsøgsdeltagernes transportudgifter vil, ved længere transport, ligeledes blive dækket. Herudover er der ingen andre vederlag i forsøget.

1. Hvervning af deltagere

Den enkelte projektdeltager rekrutteres til at medvirke i forsøget pr. brev. Projektdeltagere får med brevet tilsendt den skriftlige deltagerinformation og bliver efterfølgende kontaktet telefonisk af projektkoordinator, hvilket også fremgår af det tilsendte brevet.

Projektdeltagerne inviteres til at møde til en informationsdag i konferencerummet på Neuromuskulær forskningsenhed afsnit 3342, Rigshospitalet. På informationsdagen overleveres den mundtlige projektinformation og forsøgsdeltagernes rettigheder som forsøgspersoner i et sundhedsvidenskabeligt forskningsprojekt. Deltagerne vil i denne anledning have mulighed for at få besvaret eventuelle spørgsmål de måtte have i forbindelse med forsøget. Hvis forsøgsdeltagerne herefter ønsker at deltage i forsøget underskrives den skriftlige samtykkeerklæring og der aftales en dato til afholdelse af første forsøgsdag. Forsøgsdeltagere har en uges betænkningstid efter den mundtlige information, til at bestemme om de vil medvirke i forsøget, hvilket også vil fremgå af informationen.

Studiet anmeldes til Videnskabsetisk Komité. Al korrespondance arkiveres af den projektansvarlige. Gældende regler for information og samtykke af patienten, samt beskyttelse af patientdata vil blive overholdt. Der indhentes såvel mundtligt som skriftligt samtykke fra patienten

1. Tilgængeligheden af oplysninger

Oplysning om projektet kan indhentes hos stud.med. Christoffer Rasmus Vissing, afsnit 3342 Rigshospitalet. Tlf. 3545 6135, eller mail christoffervi@gmail.com.

1. Offentliggørelse af forsøgsresultater

Forsøgsresultaterne, både positive, negative og inkonklusive fund, vil hurtigst muligt efter forsøgets afslutning blive bearbejdet og udgivet i et videnskabeligt tidsskrift.

1. Videnskabsetisk redegørelse

Forsøget vil belyse om fysisk træning kan forbedre konditions- og funktionsniveau hos patienter med muskelsvind.

Forsøget kræver at forsøgsdeltagerne får foretaget konditionstests (max-test, gang-test, trappe-test og stol til stand-test), muskelstyrke-test og blodprøver.

Selve cykelforsøget kan medføre ubehag i forbindelse med fysisk aktivitet under selve testen. Der er desuden en lille risiko for udvikling af kardielle symptomer, muskelsmerte og -skade forbundet med cykeltesten. Dette forebygges under forsøget ved monitorering af puls og visuel udtrætningsskala (Borg).

Gang-, trappe-, og stol til stand-testene medfører alle en risiko for ubehag ved muskeludtrætning. Blodprøvetagning betragtes som et rutineindgreb med minimal infektionsrisiko. Dog viser tidligere erfaringer at nogle forsøgspersoner finder det ubehageligt. Vi har aldrig observeret infektion, der har skyldtes blodprøvetagning.

Vurdering af muskelstyrke er ikke til gene for patienterne.

Der findes på baggrund af ovenstående ikke etiske problemer i forsøget.

1. Oplysning om erstatnings- eller godtgørelsesordninger

Forsøget udføres under direkte ansvar af Rigshospitalet og er dækket af Patientforsikringsordningen.

Retningslinjer for mundtlig deltagerinformation.

Forsøgspersoner inviteres per brev til at deltage i forsøget. Vedhæftet brevet er den skriftlige deltagerinformation, et ark, der beskriver deltagernes rettigheder og en invitation med en dato for den mundtlige information. Forsøgspersonerne vil i invitationen blive opfordret til at tage kontakt til projektkoordinator telefonisk eller per mail, for at besvare om de ønsker, at deltage i forsøget eller ej. Ved manglende svar, vil projektkoordinator rette henvendelse til forsøgsdeltageren telefonisk.

Den mundtlige deltagerinformation kommer til at foregå i et uforstyrret lokale på Neuromuskulær forskningsenhed, Rigshospitalet. Forsøgspersonen er før ankomsten på Neuromuskulær forskningsenhed underrettet om sin ret til at have en bisidder med ved informationssamtalen.

Efter den mundtlige deltagerinformation på Neuromuskulær forskningsenhed har forsøgspersonerne syv dages betænkningstid til at finde ud af om de vil deltage i forsøget. Hvis forsøgsdeltageren samtykker på dagen for den mundtlige deltagerinformation, kan datoen for den første forsøgsdag arrangeres umiddelbart efter at forsøgsdeltageren har modtaget en kopi af den skriftlige samtykkeerklæring.

Den mundtlige information gives på Neuromuskulær forskningsenhed af stud.med. Christoffer Rasmus Vissing og vil inkludere en gennemgang af forsøgspersonens rettigheder.
