# Supplementary material for: Aerobic Training in Patients with Congenital Myopathy
Source: PLoS One. 2016 Jan 11;11(1):e0146036. doi: 10.1371/journal.pone.0146036 (PMC4709049; doi:10.1371/journal.pone.0146036)
Supplement: S3 Text — (DOCX) [file pone.0146036.s003.docx]

Trial study protocol

The effect of a bike-training program in patients with muscle diseases

*- Translated from Danish by Gitte Hedermann -*

*Approved by the Regional Committee on Health Research Ethics of the Capital Region of Denmark on June 17, 2013*

1. Aim and background

Background

Muscular dystrophies are a group of hereditary diseases known by a progressive loss of muscle mass and strength in the patients who are affected. This is all due to defects in proteins important to the function of the muscle cells.

Muscular dystrophies are congenital disorders. The phenotypes are similar since they all involve skeletal muscle and leads to a progressive loss in muscle strength. The debut of the disease and the progression varies from the different subtypes.

Patients with muscular dystrophies have higher levels of creatine kinase as a marker of on-going damage to the muscle cells. On MRI these patients will have high levels of fat in the muscles.

**Training**

The effects of training in healthy people and in patients with chronic diseases have been studied throughout the years with great interests.

Until a few years ago it was not recommended that patients with muscular dystrophy should train. Some believed that training would accelerate the breakdown of muscle cells. Different clinical trials have since proved that physical training, and especially aerobic training, is a safe and effective treatment of a number of different muscle disorders. The effect has not been studied in all types of muscular dystrophy – and there is only a few studies that have investigated the effect of the training program over a longer period than a couple of months.

Aim

A person’s maximal oxygen capacity (VO_2max_) is a clinical estimate for a person’s fitness. It is defined by the person’s capability to absorb, circulate and use oxygen – and thereby their capability to do physical work.

We will investigate if a bike-training program of 10 weeks can improve fitness and daily function of living in patients with muscular dystrophy. We will investigate if the effects of training can be maintained with continues training over a year. We will also include patients sitting in wheelchairs and train them under special conditions.

The effect of aerobic training is of special interest in patients with muscular dystrophy since mutations in the proteins of the muscles can lead to increased sensitivity due to mechanical stress.

The effect of the training program is evaluated by clinical parametres: maximal oxygen capacity (VO_2max_), heart frequency (HR_max_) and workload (W_max_). We will measure the level of creatine kinase (CK) in plasma. The patients will do a muscle strength test, fill out questionnaires and do some functional tests. The functional tests will be a 6-minute-walk test, a sit-to-stand test and a stair-test. In the group of muscular dystrophy patients who are wheelchair users, the parameters we measure will be a bit different.

There are no curative treatments available in the majority of inherited muscle diseases. It would therefore be of great importance to evaluate if the physical training has a clinical use as a treatment tool in this group of patients.

**Primary outcome measures**

- Fitness test with determination of VO_2max_ and/or W_max_ on an exercise bike.

**Secondary outcome measures**

- The three functional tests that show trial participants functionality before and after the training period.
- Muscle strength at inclusion.
- Attempt participants CK values that tells something about muscle injury before, during and after the trial.
- Self-rated improvement in muscle function

1. Methods

Subjects

The experiment is performed as a 10-week long bike-training program in their own homes, where patients with genetically verified muscle diseases bike 30 minutes at a time every other day or at least three times a week.

If patients are found to have a beneficial effect of aerobic training, they will be offered to continue in an almost identical monitored exercise program that will run for a year.

The participants will be invited to participate in the trial per letter. It is expected that the trial will include 7-9 subjects for each sub-group between the ages 18-75 years. We plan to investigate 4-6 muscle-wasting diseases, so it is 30 to 50 subjects in total. The project seeks to include people with diseases as Bethlem myopathy, Ullrich's disease, subtypes of Limb-girdle muscular dystrophy type 2, mitochondrial muscular dystrophy and Duchenne and Becker muscular dystrophy.

The trial will include 3-5 test days on Neuromuscular Research Unit, Section 3342, Rigshospitalet. There will be two test days before the 10-week exercise program and one after the training program. If the subject wishes to continue in the trial for one year, it will include an additional test day for six months and a final test day after one year.

Training description

**Fitness test and bike-training program**

Before the training period starts, the subjects’ VO_2max_, HR_max_ and W_max_ are determined at a peak exercise test on a cycle ergometer, where the workload gradually increased to exhaustion. The test should last approximately 12-15 min.

Subsequently, the study participants are to train for 10 weeks at a stationary bike. The subjects have to cycle every other day, or at least three times a week, and training must be carried out in a fixed pulse interval corresponding to 70% of their maximal oxygen uptake. The pulse interval is monitored with a supplied pulse monitor. The training is stepped up in the trial, such that the volunteers during the first week run 10 minutes in the prescribed pulse interval, in the second week run for 20 minutes in the pulse interval, and from the third week and to run for 30 minutes in the pulse interval. The subjects should always warm up for 5 minutes at low load before running the pulse interval.

After 10 weeks of training, the study participants will again have measured VO2 max, HR max and Wmax on a peak exercise test. The results of this test are compared with the results obtained in the test immediately before the training program (second test) for possible improvement / deterioration. If the trial participant is interested to continue to train for beyond the 10 weeks, they can continue with the training program for a year, to assess the long-term effects of aerobic fitness training in muscular dystrophy patients.

The participants must attend two test days before bicycle training program initiated to ensure that the results obtained in the experiment is not determined in a learning effect from test to test.

**Muscle strength**

Muscle strength will be evaluated at inclusion by MRC score and/or handheld dynamometer.

**6 minute walk test**

The patient is to walk as far as possible on 6 minutes on a 30 meter long track, which is marked by two cones. The length is registered before and after cycling training program.

**Timed up and down stair test**

The patient starts 30 cm from a staircase with 14 steps, each measuring 19.5 cm in height. From here they should be instructed to quickly but surely to climb stairs, turn on top and return to the base in time. Patients can choose how to climb stairs. The test is performed before and after the training program with the registration of any effect, and is an expression of their functional mobility.

**Chair stand test**

The subject should sit on a chair without armrests with crossed arms and feet on the floor. During instruction, the patient should get up from the chair without using the arms / hands. If this succeeded, the patient must get up 5 times from the chair at the time. The time is recorded and the time before cycling training program comparable to the post.

**Questionnaires**

Participants must complete a questionnaire before and after the trial.

**CK-levels**

CK is a marker of the degree of muscle damage in patients. CK is determined by blood sampling on the two experimental days (week 0 and 10) at the Neuromuscular Research Unit and during bicycle training program at the Neuromuscular Research Unit or at their local doctor. If the study participants subsequently included in the part of the trial, which extends over one year, they subsequently measured CK values every three months.

Training description wheelchair-bound subjects (not translated since this is not relevant for our study)

Da de muskelsvindspatienter, der er kørestolsbundne er for svage til at cykle på en konventionel ergometercykel, skal deres træning foregå på en motoriseret trædecykel (som fx Motomed Viva 2). Den motoriserede trædecykel er opbygget således at forsøgsdeltagerne træner siddende i deres egen stol eller kørestol. Cyklens pedaler kører automatisk rundt uden hjælp fra forsøgsdeltageren, men er i stand til at måle hvor stor en kraft brugeren bidrager med.

Som de andre forsøgsdeltagere skal brugerne af denne cykel deltage i tre testdage, træne i 10 uger (også pulsmonitoreret hver anden dag og 35 minutter af gangen) og have foretaget tre blodprøver efter henholdsvis 2, 4 og 7 uger. Kørestolsbundne forsøgsdeltagere kan af praktiske grunde ikke udføre de tre funktionstests eller få målt deres VO_2max_, hvorfor det endelige effektmål på træningseffekten er ændringen i hvor stor en kraft de kan træde med (W_max_).

1. Blood samples

During the trial a number of blood samples will be taken for determination of muscle enzyme CK. The blood samples are to ensure that there is no muscle damage during training. A blood sample is 5 ml of blood, corresponding to 1% of what will be given during a normal dispensing by a blood donor. There is always a minimum risk of infection by blood sampling. This will be prevented by alcohol cleaning of the injection site. The blood will be analysed immediately after it is taken and any remainder will destroyed.

1. Statistical considerations and strength calculation

The statistical calculations are based on previous studies that have shown efficacy of training patients with muscular dystrophy. The following strength calculation is based on the need to prove an effect among each muscle dystrophy subtype separately. The figures in the strength calculation is based on the results we have achieved in a number of similar training trials of muscular dystrophy patients, as we have previously made.

Strength calculations:

(z_1-α/2_ + Z_1-β_ )^2^ * SD^2^

n ≥ _________________________

d^2^

Z_1-α/2_ = 1.96; risk of type 1 error: (α=0.05)

Z_1-β_ = 1.28; risk of type 2 error: (1-β) = 90%

SD = 0,152;

d = 0,186; (MIREDIF) Improvement in VO_2max_.

Dvs.

(1.96 + 1.28 )^2^ * (0,152)^2^

n ≥ _________________________ = 7

(0,186)^2^

Appropriate descriptive statistics (means, SD, median and range) will be used.

Differences between before and after the training will be tested using a paired t-test.

Additional relevant statistics will be used as needed.

The part of the study dealing with the wheelchair-bound muscular dystrophy patients can be considered as being of a more exploratory nature.

Based on the strength calculation, we seek to include 7-9 muscular dystrophy patients from each subgroup with a total number of 50 subjects.

1. Inclusion and exclusion criteria

Inclusion criteria

Danish patients diagnosed with muscular dystrophy.

Exclusion criteria

Patients deemed too physically weak / weakened to carry 10-week training program.

Patients with competitive diseases that will influence the interpretation of the efficacy of physical training. This also include patients, who are pregnant or suffers from cardiovascular diseases.

Patients who cannot mentally cooperate.

Subjects

The project will include subjects aged 18-75 years, and both women and men can be included. It is estimated that 7-9 subjects are included per subgroup. In total about 50 patients.

1. Side effects, risks and disadvantages

The study includes an invasive procedure in the form of blood sample. There is always a minimal risk of infection that is prevented by alcohol cleaning of the injection site. Some patients are nervous and anxious before testing. This is prevented by thorough information and by treating the patient in a calm way. The bike trial involves risk of developing heart problems. Also discomfort associated with the hard physical exertion of the subjects. In addition, there is a risk that patients will have muscle pain and possible. Muscle injury. All this will be attempted prevented by monitoring heart rate and visual pain (Borg scale) during the test.

There is also a known risk of muscle damage associated with training individuals with highly elevated CPK value. This will be prevented by informing patients about the danger signs, which consists of muscle pain, by monitoring CK levels during the experiment and by the project leaders that will have on-going contact with the subjects throughout the study. Patients are also given a training manual that describes in detail normal and abnormal side effects of physical training, and a list of danger signs, they should be aware of, during and after exercise. If the patients in the trial experience intolerable side effects as a result of endurance training, they will be dragged out immediately out of the trial. Patients can call the project group any time during the trial.

1. Respect for the subjects' physical and mental integrity and privacy

The information concerning the individual subject is protected under the Act on processing of personal data and health law. The project reported to the Data Protection Agency.

1. Economy
2. The project was undertaken by professor John Vissing in collaboration with medical student, Christoffer Rasmus Vissing.
3. The project is supported by Rigshospitalet (daily operations). The aid will be paid by Rigshospitalet to cover operating expenses, bikes, heart rate monitors and the like.
4. There is no financial conflict of interest, and the investigator has no financial or other links to the private companies, which should have interests in the project described.
5. Relevant clause in the contract between sponsor and place of trial

Professor John Vissing is responsible for the trial. No relevant clause between partners.

1. Remuneration or other benefits

All subjects will be offered lunch after completion of the test day and the test participants' travel costs will, at long transport, also be covered. In addition, there is no other remuneration in the trial.

1. Recruitment of participants

The subjects are recruited to participate in the experiment by letter. Subjects will receive a letter with written information for participants and is subsequently contacted by telephone.

Project participants are invited to attend an information day in the conference room at the Neuromuscular Research Unit Section 3342, Rigshospitalet. At the information day they will receive an oral project information and information about trial participants' rights as subjects in a health science research. Participants will on this occasion be able to ask any questions they may have in connection with the trial. If the study participants then wish to participate in the study, they will have to sign the written consent form and agreed a date for the holding of the first test day.

The study is reported to the Research Ethics Committee. All correspondence filed by the person responsible. Applicable rules for information and consent of the patient, and the protection of patient data will be respected. There will be obtained oral and written consent from the patient.

1. The availability of information

Information on the project can be obtained from medical student, Christoffer Rasmus Vissing, Section 3342 Rigshospitalet. Tel. 3545 6135, or mail christoffervi@gmail.com.

1. Publication of test results

The test results, positive, negative and inconclusive findings will as soon as possible after completion of the trial be processed and published in a scientific journal.

1. Research Ethics statement

The trial will shed light on if physical exercise can improve fitness and functional capacity in patients with muscular dystrophy.

The experiment requires that trial participants undergo fitness tests (max test, walk test, stair test and chair stand test), muscle strength tests and blood tests.

The bike training can cause discomfort associated with physical activity during the test. There is a small risk of the development of cardiac symptoms, muscle pain and damage associated with the cycling test. This prevented during the trial by monitoring heart rate and visual scale (Borg).

Walking, stair and chair to stand tests leads all risk of discomfort with fatigue. Blood considered a routine procedure with minimal risk of infection. However, past experience shows that some subjects find it uncomfortable. We have never observed infection.

Assessment of muscle strength is not a nuisance to the patients.

Found on the basis of the above there is non-ethical problems in the experiment.

1. Information about replacement or reimbursement schemes exist

The experiment is performed under the direct responsibility of Rigshospitalet and is covered by the Patient Insurance Scheme.

Guidelines for oral information for participants.

Subjects are invited by letter to participate in the trial. Attached to the letter is the written participant information, a sheet describing the rights and an invitation to a date for the oral information. The subjects will in the invitation be encouraged to contact the project coordinator by telephone or by mail, to answer whether they want to participate in the trial or not. In the absence of a response, the project coordinator will contact the trial participant by telephone.

The oral information for participants will take place in an undisturbed room of Neuromuscular Research Unit, Copenhagen University Hospital. The subject is informed of his right to have an observer present at the interview before the arrival at the Neuromuscular Research Unit.

After the oral information on the Neuromuscular Research Unit the subjects have seven days of reflection to find out whether they will participate in the trial. If the trial participant consents on the day of the oral participant information, the date of the first test day is arranged immediately after the trial participant has received a copy of the written consent form.

The oral information given at the Neuromuscular Research Unit will include a review of the subject's rights.
